# Comprehensive genomic profiling identifies a novel *TNKS2–PDGFRA* fusion that defines a myeloid neoplasm with eosinophilia that responded dramatically to imatinib therapy

**DOI:** 10.1038/bcj.2014.95

**Published:** 2015-02-06

**Authors:** Z R Chalmers, S M Ali, R S Ohgami, P V Campregher, G M Frampton, R Yelensky, J A Elvin, N A Palma, R Erlich, J-A Vergilio, J Chmielecki, J S Ross, P J Stephens, R Hermann, V A Miller, C R Miles

**Affiliations:** 1 Foundation Medicine Inc., Cambridge, MA, USA; 2 Department of Pathology, Stanford School of Medicine, Stanford, CA, USA; 3 Clinical Laboratory, Hospital Israelita Albert Einstein, Sao Paulo, Brazil; 4 Department of Pathology, University of Michigan, Ann Arbor, MI, USA; 5 Department of Pathology and Laboratory Medicine, Albany Medical Center, Albany, NY, USA; 6 Northwest Georgia Oncology Centers, Jasper Cancer Center, Jasper, GA, USA

Myeloid and lymphoid neoplasms with *PDGFRA* gene rearrangements are a category of rare diseases that typically manifest with peripheral blood eosinophilia accompanied by eosinophilic tissue infiltrates, and may be associated with typical or atypical mast cell proliferations. These tumors can present as myeloproliferative neoplasms, acute myeloid leukemias or lymphoblastic leukemias/lymphomas and are, consequently, classified within the larger category of ‘Myeloid and lymphoid neoplasms with eosinophilia and abnormalities of *PDGFRA*, *PDGFRB* or *FGFR1'* in the 2008 World Health Organization (WHO) classification. For neoplasms harboring *PDGFRA* rearrangements, *FIP1L1–PDGFRA* is by far the most common fusion detected, reflecting an 800-kb intrachromosomal deletion [del(4)(q12q12)].^1^

Identification of *PDGFRA* rearrangement is both an important diagnostic and predictive marker, as these neoplasms are typically responsive to treatment with imatinib.^2, 3^ The atypical mast cell proliferations that accompany these tumors may result in a mistaken diagnosis of systemic mastocytosis (SM),^4, 5^ which is associated with an activating D816V alteration in *KIT.*^6^ The discrimination of these entities is important, as *PDGFRA*-rearranged tumors with eosinophilia, unlike SM harboring *KIT* D816V, are typically imatinib responsive.^7^

We describe a novel *TNKS2–PDGFRA* fusion in a myeloid neoplasm with eosinophilia, detected via comprehensive genomic profiling with a next-generation sequencing-based assay (FoundationOne), the presence of which correlated with the results from multiprobe fluorescent *in situ* hybridization (FISH) testing. Initially this patient was diagnosed with an aggressive SM (aSM); however, detection of this novel fusion, a translocation between chromosomes 4(q12) and 10(q23.3), resulted in diagnostic reclassification and led to targeted drug therapy with a resulting dramatic clinical response.

A 58-year-old Caucasian woman presented with left upper quadrant pain and hepatosplenomegaly with the splenic tip palpable to 15 cm below the left costal margin and liver palpable 5 cm below the right costal margin, with 6 years of chronic, untreated hepatitis C and hepatic cirrhosis diagnosed 2 years prior. An abdominal magnetic resonance imaging scan confirmed splenomegaly with the evidence of splenic infarct and a nodular liver, consistent with cirrhosis. A small amount of ascites was present. Her medical history was significant for mild thrombocytopenia of several years duration with no evidence of bleeding.

A bone marrow core biopsy was performed and was hypercellular (100% cellularity) with dense infiltrates and aggregates of >15 spindled mast cells as well as increased eosinophils. Mast cells were positive for CD2 and CD25 expression by immunohistochemistry. Targeted genomic analysis for mutations in *KIT* was ordered, but ultimately failed due to insufficient sample material. Cytogenetic analysis was ordered but the results were not immediately available, given the inherent time needed for processing. On the basis of these findings, the patient was initially diagnosed with aSM. A peripheral blood count performed a month after initial diagnosis revealed leukoerythroblastic features with a marked absolute eosinophilia (white blood cells of 20.6 × 10^9^/l, absolute eosinophil count of 3708/μl) and no circulating blasts. An expanded blast population was not seen either in the marrow aspirate smear or using CD34 immunohistochemistry on the core biopsy. The patient's clinical status deteriorated rapidly with worsening thrombocytopenia and progressive anasarca, necessitating hospitalization. Diuretics were of little benefit.

Subsequent FISH testing was reported positive for a *PDGFRA* gene rearrangement and was attributed to presence of the canonical *FIP1L1–PDGFRA* fusion. However, karyotyping identified a translocation involving chromosomes 4 and 10. Comprehensive genomic profiling by FoundationOne performed on a bone marrow aspirate fully characterized the presence of a t(4:10) abnormality, identified as a novel fusion of *TNKS2 and PDGFRA* (Figure 1). Further, a *KRAS* G12D point mutation was detected. These findings resulted in reclassification of this disease from aSM to a myeloid neoplasm with eosinophilia harboring a *PDGFRA* rearrangement.

On the basis of this diagnosis, the patient was started on a course of imatinib (400 mg, daily) and the clinical benefit was dramatic with immediate onset of diuresis, resolution of the anasarca, reduction in splenomegaly and steady improvement in the platelet count.

At the time of publication, the patient remains on a maintenance dose of imatinib (100 mg, daily) and a subsequent bone marrow biopsy 4 months after initiation of therapy revealed a dramatic normalization in marrow cellularity without eosinophilia or mast cell proliferation. Follow-up multiprobe FISH for *PDGFRA* abnormality at this time was negative.

A bone marrow aspirate was collected via needle biopsy and comprehensive genomic profiling (FoundationOne) was performed in a Clinical Laboratory Improvement Amendments-certified laboratory, College of American Pathologists and New York State accredited (Foundation Medicine, Cambridge, MA, USA). Methods of the clinical cancer gene assay used to analyze this patient have been previously published and the assay performance has been validated rigorously.^9^ Here we provide a brief summary. DNA is extracted from formalin-fixed, paraffin-embedded tissue (⩾1 mm^3^) containing no less than 20% tumor nuclei by enzymatic digestion and subsequent purification. DNA is fragmented by sonication to 200 bp segments. Indexed sequencing adapters are ligated to the DNA fragments and PCR amplified to yield 500 ng of sequencing library. Hybridization selection is performed using individually synthesized baits targeting 3679 exons of 236 cancer-related genes and 47 introns of 19 genes frequently rearranged in cancer. The Illumina HiSeq 2500 (Illumina Inc, San Diego, CA, USA) platform is used in 49 × 49 paired-end sequencing. Sequence data are mapped to the human genome (hg19) using BWA aligner v0.5.9.^10^ Sequence data are analyzed through a computational analysis pipeline to call variants present in the sample, including substitutions, short insertions and deletions, rearrangements and copy-number variants.

FISH studies were performed with a standard commercially available multiprobe assay. Red and green probes hybridize to the 3' and 5' end of *FIP1L1*, respectively. An aqua probe hybridizes to *PDGFRA*. Loss of the 3' end of *FIP1L1*, as evidenced by an absent red signal, is considered a positive result for *PDGFRA*–*FIP1L1* fusion.

We present a patient initially diagnosed as harboring aSM and ultimately reclassified most appropriately as having a myeloid neoplasm with rearrangement of *PDGFRA* with a novel fusion partner, *TNKS2*, identified by comprehensive genomic profiling. Since the first detection of *FIP1L1–PDGFRA* fusions was in 2003,^1^ many other fusion partners of *PDGFRA* have been discovered. These include translocations with chromosomes 4, 12 and 22, and partial insertions of chromosome 9.^11^ We have observed a translocation of *TNKS2* and *PDGFRA* with breakpoint in intron 25 and exon 12, respectively. The discovery of a novel fusion of *TNKS2* with *PDGFRA* further demonstrates the diversity of alterations possible in these myeloid neoplasms with eosinophilia.

From our clinical observations, it is unknown whether *TNKS2* has a role identical to that of *FIP1L1* in the context of a fusion with *PDGFRA*. Further *in vitro* studies are needed to elucidate whether the identity of the fusion partner in a *PDGFRA* rearrangement has functional and clinical implications.

Unlike the near pathognomonic *KIT* D816V alteration in SM, *FIP1L1–PDGFRA* fusions are associated with a favorable response to imatinib therapy^2, 3^ and mandate the diagnosis of a myeloid neoplasm with *PDGFRA* rearrangement. Other known fusion partners of *PDGFRA* described by Gotlib and Cools^11^ have not yet been known to respond to imatinib.^12^ The *TNKS2–PDGFRA* fusion described further extends the set of known targets for imatinib therapy. Interestingly, a concurrent *KRAS* G12D mutation was also detected in this patient. In some settings, this mutation has been associated with a poor response to tyrosine–kinase inhibition;^8^ yet the patient's response to imatinib was remarkable and has been durable to date. Others have described similar findings with crizotinib, demonstrating clinical efficacy despite the presence of concurrent *KRAS* and *MET* alterations in the same tumor.^13^

Unlike the well-characterized intrachromosomal *FIP1L1–PDGFRA* fusion of 4q12, the *TNKS2–PDGFRA* fusion observed in this patient is an interchromosomal translocation. In performing diagnostic assays, direct interrogation for the presence of *FIP1L1–PDGFRA* may not always be performed and patients are, instead, tested for deletion of *CHIC2*, a gene that lies in the region between *FIP1L1* and *PDGFRA*. This surrogate assay may be most effective for detecting recurrence of a typical *FIP1L1–PDGFRA,*^5^ but would not have resolved an interchromosomal event such as this one.

In summary, this report describes a novel *TNKS2–PDGFRA* fusion that diagnostically defines a distinct category of myeloid neoplasms with eosinophilia as defined within the WHO 2008 classification schema. Using FISH analyses, these neoplasms may be incorrectly interpreted as harboring a *FIP1L1–PDGFRA* rearrangement or may even be interpreted as fusion negative if the fusion is complex and FISH probes do not fully border the regions of interest. Identification of a *PDGFRA* rearrangement is important for both diagnosis and therapy as these neoplasms are responsive to tyrosine kinase inhibitors. However, the implications for durability of therapeutic response and prognosis, particularly with respect to the specific *PDGFRA* fusion partner, are not yet known. Comprehensive genomic profiling offers an accurate and detailed analysis platform that reliably detects the complex interchromosomal events at gene-level resolution.

## Figures and Tables

**Figure 1:**
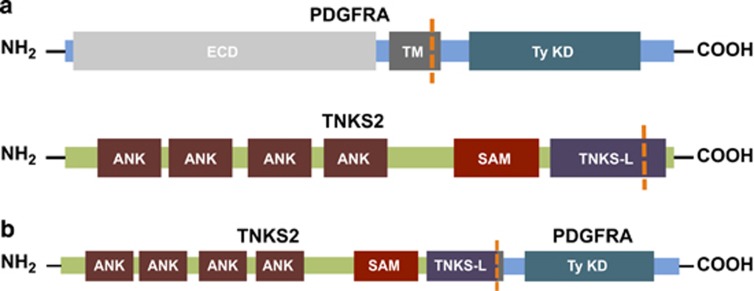
Intrachromosomal rearrangement of *TNKS2* and *PDGFRA* results in a likely fusion gene. ANK, ankyrin domains; ECD, extracellular domain; SAM, sterile alpha motif; TM, transmembrane; TNKS-L, tankyrase-like; TyKD, kinase domain.

## Notes

ZRC, SMA, GMF, JSR, RY, NAP, JAE, JC, PJS, VAM, RE and PVC are employed by and have equity interest in Foundation Medicine, Inc. The remaining authors declared no conflict of interest.

## References

[bib1] Cools J DeAngelo DJ Gotlib J Stover EH Legare RD Cortes J A tyrosine kinase created by fusion of the PDGFRA and FIP1L1 genes as a therapeutic target of imatinib in idiopathic hypereosinophilic syndrome N Engl J Med 2003 348 1201 1214 12660384 10.1056/NEJMoa025217

[bib2] Pardanani A D'Souza A Knudson RA Hanson CA Ketterling RP Tefferi A Long-term follow-up of FIP1L1-PDGFRA-mutated patients with eosinophilia: survival and clinical outcome Leukemia 2012 26 2439 2441 22705991 10.1038/leu.2012.162

[bib3] Jain N Cortes J Quintás-Cardama A Manshouri T Luthra R Garcia-Manero G Imatinib has limited therapeutic activity for hypereosinophilic syndrome patients with unknown or negative PDGFRα mutation status Leuk Res 2009 33 837 839 19013640 10.1016/j.leukres.2008.10.004 PMC4422052

[bib4] Tefferi A Verstovsek S Pardanani A How we diagnose and treat WHO-defined systemic mastocytosis in adults Haematologica 2008 93 6 9 18166778 10.3324/haematol.12324

[bib5] Pardanani A Ketterling RP Brockman SR Flynn HC Paternoster SF Shearer BM CHIC2 deletion, a surrogate for FIP1L1-PDGFRA fusion, occurs in systemic mastocytosis associated with eosinophilia and predicts response to imatinib mesylate therapy Blood 2003 102 3093 3096 12842979 10.1182/blood-2003-05-1627

[bib6] Garcia-Montero AC Jara-Acevedo M Teodosio C Sanchez ML Nunez R Prados A KIT mutation in mast cells and other bone marrow hematopoietic cell lineages in systemic mast cell disorders: a prospective study of the Spanish Network on Mastocytosis (REMA) in a series of 113 patients Blood 2006 108 2366 2372 16741248 10.1182/blood-2006-04-015545

[bib7] Ma Y Zeng S Metcalfe DD Akin C Dimitrijevic S Butterfield JH The c-KIT mutation causing human mastocytosis is resistant to STI571 and other KIT kinase inhibitors; kinases with enzymatic site mutations show different inhibitor sensitivity profiles than wild-type kinases and those with regulatory-type mutations Blood 2002 99 1741 1744 11861291 10.1182/blood.v99.5.1741

[bib8] Miranda C Nucifora M Molinari F Conca E Anania MC Bordoni A KRAS and BRAF mutations predict primary resistance to imatinib in gastrointestinal stromal tumors Clin Cancer Res Off J Am Assoc Cancer Res 2012 18 1769 1776 10.1158/1078-0432.CCR-11-2230 22282465

[bib9] Frampton GM Fichtenholtz A Otto GA Wang K Downing SR He J Development and validation of a clinical cancer genomic profiling test based on massively parallel DNA sequencing Nat Biotechnol 2013 31 1023 1031 24142049 10.1038/nbt.2696 PMC5710001

[bib10] Li H Durbin R Fast and accurate short read alignment with Burrows-Wheeler transform Bioinformatics 2009 25 1754 1760 19451168 10.1093/bioinformatics/btp324 PMC2705234

[bib11] Gotlib J Cools J Five years since the discovery of FIP1L1-PDGFRA: what we have learned about the fusion and other molecularly defined eosinophilias Leukemia 2008 22 1999 2010 18843283 10.1038/leu.2008.287

[bib12] Stover EH Chen J Folens C Lee BH Mentens N Marynen P Activation of FIP1L1–PDGFRalpha requires disruption of the juxtamembrane domain of PDGFRalpha and is FIP1L1-independent Proc Natl Acad Sci USA 2006 103 8078 8083 16690743 10.1073/pnas.0601192103 PMC1472432

[bib13] Palma NA Ali SM O'Connor J Dutta D Wang K Soman S Durable response to crizotinib in a MET-amplified, KRAS-mutated carcinoma of unknown primary Case Rep Oncol 2014 7 503 508 25232318 10.1159/000365326 PMC4164090

